# Application of Supervised Machine Learning to Recognize Competent Level and Mixed Antinuclear Antibody Patterns Based on ICAP International Consensus

**DOI:** 10.3390/diagnostics11040642

**Published:** 2021-04-01

**Authors:** Yi-Da Wu, Ruey-Kai Sheu, Chih-Wei Chung, Yen-Ching Wu, Chiao-Chi Ou, Chien-Wen Hsiao, Huang-Chen Chang, Ying-Chieh Huang, Yi-Ming Chen, Win-Tsung Lo, Lun-Chi Chen, Chien-Chung Huang, Tsu-Yi Hsieh, Wen-Nan Huang, Tsai-Hung Yen, Yun-Wen Chen, Chia-Yu Chen, Yi-Hsing Chen

**Affiliations:** 1 Division of Allergy, Immunology and Rheumatology, Taichung Veterans General Hospital, Taichung 40705, Taiwan; bagidr1@hotmail.com (Y.-D.W.); yencwu@vghtc.gov.tw (Y.-C.W.); cactus3854@vghtc.gov.tw (C.-C.O.); wen76719@vghtc.gov.tw (C.-W.H.); jhjean@vghtc.gov.tw (H.-C.C.); yingchieh@vghtc.gov.tw (Y.-C.H.); blacklark@gmail.com (Y.-M.C.); zuyihsieh@gmail.com (T.-Y.H.); gtim5555@yahoo.com (W.-N.H.); mi552296@gmail.com (T.-H.Y.); minibasketball20@gmail.com (Y.-W.C.); 2 Department of Computer Science, Tunghai University, Taichung 407224, Taiwan; rickysheu@thu.edu.tw (R.-K.S.); winston@thu.edu.tw (W.-T.L.); ccwhung@vghtc.gov.tw (C.-C.H.); g08350007@thu.edu.tw (C.-Y.C.); 3 AI Center, Tunghai University, Taichung 407224, Taiwan; d09725001@ntu.edu.tw; 4 Department of Medical Research, Taichung Veterans General Hospital, Taichung 40705, Taiwan; 5 Rong Hsing Research Center for Translational Medicine, National Chung Hsing University, Taichung 402, Taiwan; 6 Ph.D. Program in Translational Medicine, National Chung Hsing University, Taichung 402, Taiwan; 7 Faculty of Medicine, National Yang-Ming University, Taipei 11221, Taiwan; 8 College of Engineering, Tunghai University, Taichung 407224, Taiwan; lunchi@thu.edu.tw; 9 Computer & Communication Center, Taichung Veterans General Hospital, Taichung 407224, Taiwan; 10 Department of Medical Education, Taichung Veterans General Hospital, Taichung 40705, Taiwan

**Keywords:** Hep-2 cell, ICAP, artificial intelligence, antinuclear antibody, machine learning

## Abstract

Background: Antinuclear antibody pattern recognition is vital for autoimmune disease diagnosis but labor-intensive for manual interpretation. To develop an automated pattern recognition system, we established machine learning models based on the International Consensus on Antinuclear Antibody Patterns (ICAP) at a competent level, mixed patterns recognition, and evaluated their consistency with human reading. Methods: 51,694 human epithelial cells (HEp-2) cell images with patterns assigned by experienced medical technologists collected in a medical center were used to train six machine learning algorithms and were compared by their performance. Next, we choose the best performing model to test the consistency with five experienced readers and two beginners. Results: The mean F1 score in each classification of the best performing model was 0.86 evaluated by Testing Data 1. For the inter-observer agreement test on Testing Data 2, the average agreement was 0.849 (κ) among five experienced readers, 0.844 between the best performing model and experienced readers, 0.528 between experienced readers and beginners. The results indicate that the proposed model outperformed beginners and achieved an excellent agreement with experienced readers. Conclusions: This study demonstrated that the developed model could reach an excellent agreement with experienced human readers using machine learning methods.

## 1. Introduction

Screening of autoantibodies using the indirect immunofluorescence (IIF) assay on human epithelial cells (HEp-2) is an essential diagnostic tool and is the standard golden method for systemic autoimmune rheumatic diseases (SARD). The HEp-2 IIF pattern reveals clinically relevant information giving direction to follow-up testing for antigen-specificity [1]. The consensus nomenclature and representative 29 patterns are established and available online at the International Consensus on ANA Patterns (ICAP) website: www.ANApatterns.org [2]. The patterns were divided into competent level versus expert level, with the intention that ANA readers should be trained to recognize all competent level patterns minimally. Clinicians should be aware of the clinical suspicion of individual patients and therefore should order second-level test by chemiluminescence, enzyme-linked immunosorbent assay (ELISA) or immunoblot according to Hep-2 IIF patterns. However, the IIF method has some unfavorable features, such as labor-intensive, time-consuming for visual evaluation, inter-observer variability [3], heavy dependence on the experience and expertise of the technologist, physician, or immunologist conducting the test.

Currently, there are different commercial systems for automated ANA IIF testing aimed at reducing hands-on labor time and good concordance with technologist ANA IIF pattern recognition has been achieved [4]. Nevertheless, a number of weaknesses still exist. First, the automated HEp-2 cell classification used in previous studies usually has only six to eight classes that do not meet the ICAP’s competent-level standard. The ICAP competent-level reporting consists of 11 different patterns, namely, nuclear homogeneous (AC-1), nuclear dense fine speckled (AC-2), centromere (AC-3), nuclear speckled (AC-4, 5), discrete nuclear dots (AC-6, 7), nuclear nucleolar (AC-8, 9, 10), cytoplasmic fibrillar (AC-15, 16, 17), cytoplasmic speckled (AC-18, 19, 20), cytoplasmic reticular/mitochondrion-like (AMA) (AC-21), cytoplasmic Golgi (AC-22), cytoplasmic rods and rings (AC-23). Second, previous studies usually only analyzed the features of interphase cells. Nevertheless, according to the ICAP standard, scientists must identify the features of both interphase cells and mitotic metaphase cells to determine the final patterns comprehensively. Third, most existing automated interpretation instruments have not been able to recognize mixed patterns accurately. However, in clinical daily practice, mixed patterns are prevalent in the ANA IIF test. For the above reasons, most laboratories still rely heavily on manual interpretation under fluorescent microscopes [5].

### 1.1. Related Work

Since 2002 [6], several ongoing efforts have attempted to develop a computer-assisted classification of HEp-2 IIF patterns. Different methods have been proposed, especially during the HEp-2 cell classification competitions [7,8,9]. Recently, deep convolutional neural networks (CNNs) have demonstrated outstanding performance for generic visual recognition tasks. Donato et al. [10,11] suggested to use nine pre-trained CNN models for feature extraction and classification for six different HEp-2 image patterns. The proposed methods have been applied to classification on benchmark datasets for HEp-2 cells. In the literature review, two main tasks were conducted: cell-level HEp-2 image classification (CL-HEP2IC) and specimen-level HEp-2 image classification (SL-HEP2IC). The themes of CL-HEP2IC and SL-HEP2IC were first introduced in the International Conference on Pattern Recognition ICPR 2013 and ICPR 2014 IIF image classification competitions, respectively, and then also used in ICPR 2016 competition. At that moment, the developed works were based on the public dataset. The public dataset mentioned in these papers mainly include the following four: ICPR 2012 dataset, SNPHEp-2 dataset, 13A dataset, and AIDA dataset.

The ICPR 2012 dataset, also known as MIVIA HEp-2 images dataset [12], has 1455 individual cell images annotated by immunology experts at cell level from specimen images. Each cell image is classified into one of the following six classes: homogeneous, coarse speckled, fine speckled, nucleolar, centromere, and cytoplasmic.

The SNPHEp-2 dataset is another public dataset used in previous works, which has 1884 individual monochrome cell images [13]. Each cell image is classified into one of the following five classes: homogeneous, coarse speckled, fine speckled, nucleolar, and centromere. Compared with the ICPR 2012 dataset, the SNPHEp-2 dataset is less used in the existing HEP2IC method.

The I3A dataset is one of the commonly used datasets in current literature. There exist two versions of this dataset, namely, Task-1 and Task-2 datasets. Task-1 is primarily designed for CL-HEP2IC and Task-2 for SL-HEP2IC. The Task-1 training set has 13,596 monochrome single-cell images extracted from 83 specimens using the bounding box annotations by experts. The images are divided into one of the following six classes: homogeneous, speckled, nucleolar, centromere, nuclear membrane, and Golgi. The Task-2 dataset has a total of 1008 images, and they are taken from four different locations of 252 specimen samples. Each specimen image belongs to one of the following seven classes: homogeneous, speckled, nucleolar, centromere, Golgi, nuclear membrane, and mitotic spindle.

The autoimmunity: diagnosis assisted by computer (AIDA) dataset [14] is a large-scale HEp-2 image dataset proposed as part of the AIDA project. The number of images in this dataset reached 14,393 with a variety of more-than-twenty staining patterns reported by human experts, but it is only available to AIDA project partners. Unlike the datasets mentioned above, it also contains a variety of single and mixed pattern images. The distribution of IIF patterns in the AIDA database were as followed: homogenous + nucleolar (4%), speckled + nuclear membranous (2%), homogenous (19%), fine speckled (15%), coarse speckled (11%), homogenous + speckled (8%), nucleolar (6%), speckled + nucleolar (5%), centromere (4%), others (26%). The AIDA database’s public part consists of 2080 images, including 1498 positive fluorescence intensity images and 582 negative images. Among the images with positive fluorescence, those relating to patterns belonging to the six classifications are a total of 220 single pattern images (21 homogenous, 42 speckled, 26 centromere, 62 nucleolar, 46 nuclear dots, 23 nuclear membrane) and used as testing images.

For the CL-HEP2IC, in 2018, Lei et al. [15] proposed an effective training strategy by cross-modal transfer learning to successfully train deep networks with small datasets and achieves the best mean class accuracy (MCA) of 97.14% by ResNet-50-3DT on ICPR2012 dataset and 98.42% by ResNet-50-3DCT on 13A-Task1 dataset. In 2019, Rodrigues et al. [8] compared five CNNs architectures, LeNet-5, AlexNet, Inception-V3, VGG-16, and ResNet-50, to classify HEp-2 cells on 13A-Task 1 dataset. The best MCA result was 98.28% by training the Inception-V3 model from scratch, without preprocessing and using data augmentation. Another recent work proposed by Vununu et al. [9] uses a four-stream CNN to learn local intensity and geometric information to deal with the heterogeneity problem occurring in HEp-2 cells. The proposed CNN of their work in experiment 6 achieved the MCA 98.89% on 13A-Task 1 dataset. However, according to the well-established ICAP standards, patterns discrimination needs to be comprehensively interpreted by interphase cells and metaphase cells in a specimen image but not simply based on a single interphase cell. 

For the SL-HEP2IC, in 2019, Cascio et al. [16] present an automatic HEp-2 specimen system based on a CNN method consists of a module for features extraction based on a pre-trained AlexNet network and a classification phase for the cell-pattern association using six support vector machines and a k-nearest neighbor classifier. The classification at the image-level was obtained by analyzing the pattern prevalence at cell-level. The performance analysis showed a MCA equal to 93.75% on the 13A-Task 2 dataset. A more recent work by Xie et al. [17] proposed a novel deeply supervised full convolutional network (DSFCN), which integrates the dense deconvolution layer (DDL) and hierarchical supervision structure (HS) for robust segmentation of different HEp-2 cell images and pattern classification. The performance evaluated by MCA achieved 95.40% on the 13A-Task 2 dataset, which outperforms other state-of-the-art methods.

### 1.2. Our Contributions

Some critical issues limited the above works applying to current clinical HEp-2 cell pattern recognition problems. First, the above dataset training and testing images only contain single pattern images but not mixed pattern images that are very frequent in daily work with autoimmune diseases, especially systemic lupus erythematosus. In other words, clinically, we face a HEp-2 cell multi-label classification problem instead of the multiclass classification problem seen in previous works of literature. Second, only 5–7 pattern classifications were available in previous works, which was far unmet for the standards of competent Level 11 pattern classifications.

To solve the above-mentioned clinical problems, in this work, we spent five years establishing our dataset. These positive fluorescence specimen images contain at least one pattern with various mixed pattern images (up to four patterns can overlap in an image). Experienced and certified medical technologists had labeled every image by ICAP standard with multi-label classification up to 11 patter classes: nuclear homogeneous (AC-1), nuclear dense fine speckled (AC-2), centromere (AC-3), nuclear speckled (AC-4, 5), discrete nuclear dots (AC-6,7), nuclear nucleolar (AC-8, 9, 10), cytoplasmic fibrillar (AC-15, 16, 17), cytoplasmic speckled (AC-18, 19, 20), cytoplasmic AMA (AC-21), cytoplasmic Golgi (AC-22), cytoplasmic rods and rings (AC-23). We developed machine-assisted interpretation systems using this large routine ANA dataset to develop different models by machine learning methods. The best performing model was then evaluated by inter-observer agreement (IOA) tests among experienced readers and beginners. In this paper, a publicly available pre-trained CNN model applying to a large clinically useful HEp-2 dataset with fine-tuning can achieve exceptionally high agreement of experienced human readers has been presented, even without a novel image-processing technique used. In particular, unlike almost all works presented on this topic, it meets the standards of ICAP competent level 11 classifications and mixed pattern recognition with high potential for immunologic laboratory automated diagnostic support.

## 2. Materials and Methods

### 2.1. The ANA Test 

We used the ANA IIF image dataset collected from December 2014 to March 2020 at the allergy, immunology and rheumatology division of the clinical medicine laboratory and pathological diagnosis center of Taichung Veterans General Hospital (TCVGH) in Taiwan. Clinicians requested these ANA tests in daily routine work using the automated IIF NOVA View instrument and NOVA Lite HEp-2 ANA kit (Inova Diagnostics, Inc., San Diego, CA, USA). Some patients had more than one ANA test at different time points. The NOVA View instrument consists of an automated and fully motorized IIF microscope and dual-band 40,6-diamidino-2-phenylin-dole (DAPI)/fluorescein isothiocyanate (FITC) filters, a LED light source, and a Kappa DX4 digital camera. The LED UV light source is a CoolLed PreciseExcite (CoolLED, Hampshire, UK) with excitation wavelengths of 400 nm (DAPI) and 490 nm (FITC). The NOVA View software uses DAPI fluorescence for localizing the HEp-2 cells and focusing. The image analysis is then performed based on the FITC signal. For each well in a slide, at least three images are acquired. Each cell image must contain at least 25 interphase and two mitotic (metaphase) cells in total. To meet the ICAP classification standard in our laboratory, our medical technologists manually read every sample under a fluorescent microscope.

### 2.2. Reporting of ANA Test Results 

**Figure 1:**
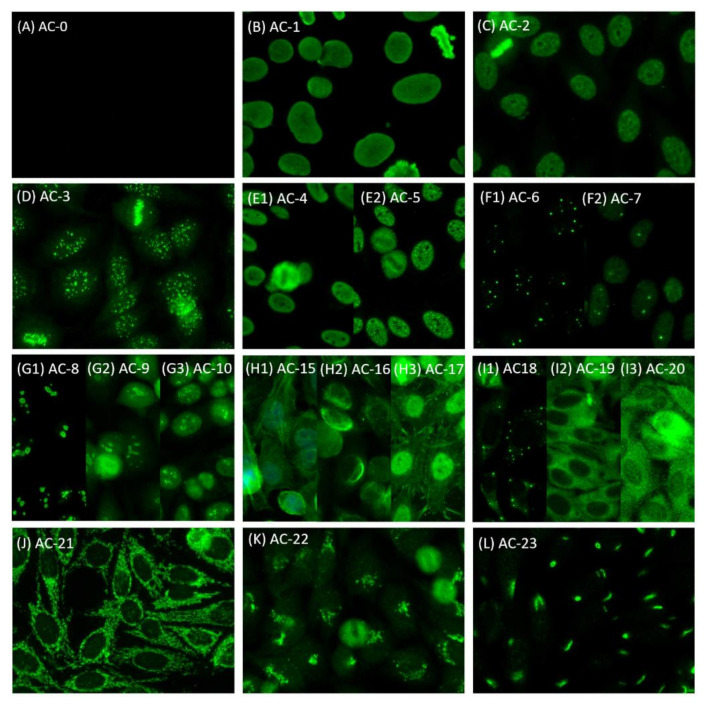
Representative images (INOVA, San Diego, USA) of human epithelial (HEp-2) cell patterns according to International Consensus on Antinuclear Antibody Patterns (ICAP) classification. (**A**) negative; (**B**) nuclear homogenous; (**C**) nuclear dense fine speckled; (**D**) nuclear centromere; (**E1**) nuclear fine speckled; (**E2**) nuclear large/coarse speckled; (**F1**) multiple nuclear dots; (**F2**) few nuclear dots; (**G1**) homogenous nucleolar; (**G2**) clumpy nucleolar; (**G3**) punctate nucleolar; (**H1**) fibrillar linear; (**H2**) fibrillar filamentous; (**H3**) fibrillar segmental; (**I1**) cytoplasmic discrete dots; (**I2**) cytoplasmic dense fine speckled; (**I3**) cytoplasmic fine speckled; (**J**) cytoplasmic reticular/mitochondrion-like (AMA); (**K**) cytoplasmic Golgi; (**L**) cytoplasmic rods and rings.

**Figure 2:**
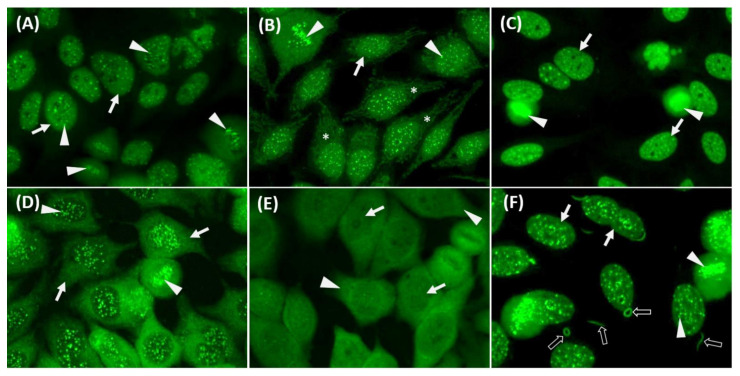
Examples of immunofluorescence mixed patterns on HEp-2 ANA images (INOVA, San Diego, CA, USA). (**A**) mixed centromere (arrowhead)and nuclear fine speckled patterns (arrow); (**B**) mixed centromere (arrowhead), nuclear fine speckled (arrow) and cytoplasmic AMA patterns (asterisk); (**C**) mixed nuclear homogenous (arrowhead) and nuclear large/coarse speckled patterns (arrow); (**D**) mixed centromere (arrowhead)and cytoplasmic speckled patterns (arrow); (**E**) mixed nuclear fine speckled (arrow) and cytoplasmic speckled patterns (arrowhead); (**F**) mixed nuclear fine speckled (arrow), centromere (arrowhead)and cytoplasmic rods and rings (black arrow) patterns.

The NOVA View system has reached a high agreement with manual microscopic reading at IIF 1: 80 screening dilution [18], so we used a dilution of 1:80 to determine the ANA patterns. In our laboratory, five experienced and certified medical technologists (considered experienced readers in this study) who had 3–12 years (mean: 8 years) of experience in ANA IIF reading were responsible for ANA pattern reporting. To ensure that high quality standards are maintained, our laboratory is evaluated using the College of American Pathologists (CAP) competence test and has been certified every year. To reach an international consensus, we adopted the classification standards expert-level reporting and interpretation principles officially published by the ICAP [2]. In mixed patterns, all nuclear patterns are reported first, followed by cytoplasmic and then mitotic patterns [19]. At least two medical technologists must have a thorough discussion for uncertain samples before issuing a final report. Clinicians would perform a second-level test by ELISA or immunoblot guided by the patient’s symptoms/physical examinations and the IIF HEp-2 pattern reports accordingly to confirm the autoantibodies. In this study, in order to allow our model to learn competent-level reporting, we attributed the minor pattern subgroups of the expert level in our raw pattern reports to the eleven main groups of the competent level. Representative images of ICAP competent-level can be viewed in the official publication [2] or on the official ICAP website. Available online: https://www.ANApatterns.org (accessed on 25 February 2021). Figure 1 shows representative classes of competent-level images and Figure 2 shows examples of mixed patterns images from our dataset.

### 2.3. Dataset Description and Imbalanced Data Correction

From 1 December 2014 to 31 December 2019, 90,109 samples with ANA IIF images were stored in our NOVA View machine. The following images were excluded: 1. Lack of formal report; 2. periodical pre-run testing samples; 3. severe abnormally exposed images; 4. performed before 1 June 2018 (our laboratory did not formally classify nuclear dense fine speckled pattern before 22 June 2017). Of the remaining 34,756 samples, 18,380 samples were categorized as negative (AC-0), which was defined as negative fluorescent staining of nuclear, cytoplasm, and mitotic cells. A total of 16.376 samples (from 11,373 patients) were categorized as positive IIF pattern(s) samples. Each sample generated 3–8 images by NOVA View instrument and these sample images were used for machine learning. Defective images were deleted from the dataset. 

For data imbalance, we collected certain patients’ serum with positive cytoplasmic fibrillar, cytoplasmic AMA, or cytoplasmic Golgi patterns for more ANA tests. Finally, we obtained 121 images with positive cytoplasmic fibrillar pattern, 74 with cytoplasmic AMA, and 406 with Golgi pattern. Due to insufficient images and no available serum, recognizing “cytoplasmic rods and rings” pattern faces the data engineering difficulties of data imbalance. In our experiments, no acceptable trained model could be conducted based on fewer data. Finally, a brightness data augmentation technique is used to generate 624 images from 54 samples (156 images) [20,21]. All pixels are augmented by 0.70, 0.85, 1.15, 1.30. After convolution filtering, the key features are extracted to train a feasible model which significantly improves the performance of cytoplasmic rods and rings pattern classification. All of the above images were used as training data to correct the problem of data imbalance.

### 2.4. Follow-up Testing for Antigen-Specificity of Different Patterns 

To verify our dataset’s clinical relevance, we analyzed the number of different patterns of relevant follow-up tests and the positive rate. The follow-up testing for antigen-specificity of different patterns were illustrated in Table 1 [1,2,22,23]. The decision to conduct follow-up testing depended on the clinicians’ judgment. We did not analyze relevant follow-up antibody tests for cytoplasmic fibrillar, cytoplasmic Golgi, and cytoplasmic rods and rings patterns as commercial kits were not available in our laboratory.

### 2.5. Data Pre-Processing

For HEp-2 IIF image information only existing in the green channel, we excluded the red and blue channels to avoid noise. Furthermore, to better extract the features and obtain a robust performance, an appropriate intensity contrast enhancement approach for image pre-processing was conducted [24]:

(1) $$I_{enhance}=\frac{I-I_{min}}{I_{max}-I_{min}}$$

where *I* is the input image, and *I*_min_ and *I*_max_ are the minima and maximum intensity values, respectively, of the input image. Using the image pixel-adjusted approach, the intensity value would normalize to equalize the scale between 0 and 1.

### 2.6. Deep Convolutional Neural Network

In this study, we selected six state-of-the-art CNN architectures for our classification issue, including VGG19, ResNet50V2, DenseNet121, MobileNetV2, Xception, and InceptionResNetV2 [25,26,27,28,29]. In addition, we transferred the weights of convolutional layers from those pre-trained models on the ImageNet dataset via the transfer learning technique and identified the one with the best performance as the proposed approach with the highest F1 score and kappa value.

We adopt six CNN architectures that are pre-trained for the large image classification tasks (ImageNet) and then fine-tune its parameters towards our ANA patterns classification problem. The initial weights of the pre-trained model were transferred for the new object classes. During the training phase, we try to optimize the learning model by setting necessary hyperparameters, including learning rate, epoch, batch_size. (With a learning rate of {1 × 10^−3^, 1 × 10^−4^, 1 × 10^−5^, 1 × 10^−6^}, batch size of {16, 24, 30}, and dropout rate of {0.4, 0.5, 0.55, 0.6}). All training processes will be stopped when meeting early stopping rules in five continuous epochs [30].

### 2.7. Training Protocol

According to the rules of ICAP, interpretation ANA patterns must consider both the interphase cell and the metaphase cell. Therefore, the input images in our study were based on specimen-level HEp-2 images rather than cell-level HEp-2 images.

**Figure 3:**
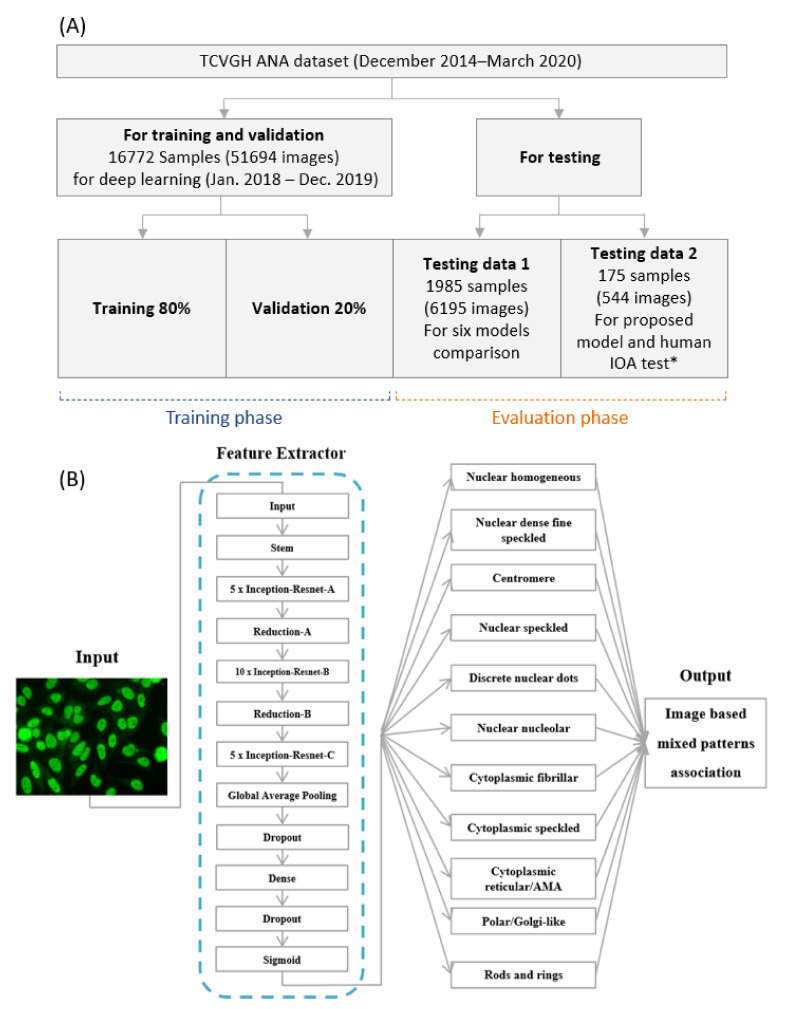
(**A**) Schematic depiction of dataset in this study for training phase and evaluation phase. (**B**) The overall convolutional neural network (CNN) architecture of the proposed model (InceptionResNet V2) for ANA mixed patterns classification. TCVGH = Taichung Veterans General Hospital; CNN = convolutional neural network; IOA = inter-observation agreement; * among five experienced-certified medical technologists, two new rheumatology fellows-in-training and the proposed model.

Hestness et al. [31] have shared the traditional leave-one-specimen-out (LOSO) procedures and suggested train-validation split ratio of 80:20. In our experiments, all images were randomly chosen and partitioned into training and validation datasets by 80:20 ratio and resized to 299 × 299 pixels. There are 16,772 samples consists of 51,694 images in total, from year 2018 to 2019 for training and validation. We used 1895 samples consists of 6195 images as Testing Data 1 from January 2020 to March 2020 for models testing. The overall flowchart is presented in Figure 3A. The networks were trained for 30 epochs and a mini-batch size of 30 on a Graphic Processing Unit (GPU) (NVIDIA TITAN V, 12GB RAM). To avoid overfitting, we stopped the training phase if the loss of validation dataset failed to improve for five epochs. These models followed the settings of parameter: Adam optimizer with an initial learning rate of 0.0001, a binary cross entropy loss function, ReLU activation function, and sigmoid activation function for the output layer. Data augmentation schemes comprising random horizontal/vertical flips were performed to increase the data size. Brightness data augmentation was particularly applied to “cytoplasmic rods and rings” pattern images for extreme insufficient original image numbers. The details of brightness data augmentation could be seen in Section 2.3. 

### 2.8. From Image Prediction to Sample Prediction

Each ANA sample yielded at least three images to ensure that there were sufficient metaphase cells identified by the NOVA view machine. As the ultimate aim was to apply supervised machine learning in clinical practice, we adopted a modified voting approach to comprehensively evaluate the predictive results of all images in each sample. For each HEp-2 cell image, a probability distribution was generated by the pre-trained model across the eleven possible classes, and classes of probability ≥0.5 were selected as image prediction. Among each sample’s images, classes supported by at least two images were adopted as the final sample prediction. Because the typical metaphase cell is the most crucial cell for identifying nuclear homogenous patterns, it may only appear in one of the images in each sample, thus, the final prediction of a nuclear homogenous pattern just needs one supporting image. Furthermore, as nuclear dense fine speckled pattern cannot coexist with either nuclear homogeneous or nuclear speckled patterns, we regarded nuclear dense fine speckled pattern as a priority and ignored the other predicted patterns if they coexisted.

### 2.9. Evaluation Protocol

We developed six pre-trained models and evaluated their performance by Testing Data 1, which consists of 1985 samples with a total of 6195 images (Figure 3A) collected from January 2020 to March 2020. Then we randomly selected 175 samples with a total of 544 images from January 2020 to March 2020 as Testing Data 2 for the inter-observer agreement test among five experienced readers, two beginners, and the best performance proposed model.

In this study, we were interested in discriminating within eleven classes following the standards of ICAP competent-level reporting. It may contain several patterns in the meantime, so it is also called ’multi-label’ in the machine learning field [32,33] and in the presence of class imbalance. Therefore, to measure and evaluate the classification performance of imbalanced data, we propose adopting the metrics in terms of precision, recall, and F1 score. For the results of the binary classifier of each ANA pattern, the confusion matrix contains the numbers of true positives (*TP*), true negatives (*TN*), false positives (*FP*), and false negatives (*FN*). The accuracy (a ratio of true positive + true negative to the total testing samples), precision (the proportion of positive predictions that are actually positive labels), recall (the proportion of positive labels that are correctly classified, also known as sensitivity), and F1 score (the weighted average performance of precision and recall) are defined as followed:

(2) $$Accuracy=\frac{TP+TN}{TP+TN+FP+FN}$$

(3) $$Precision=\frac{TP}{TP+FP}$$

(4) $$Recall=\frac{TP}{TP+FN}$$

(5) $$F1=\frac{2\times Precision\times Recall}{Precision+Recall}$$

The inter-observer agreement, determined by Cohen’s kappa, can be expressed as follows [34]

(6) $$Kappa=\frac{p_{o}-p_{c}}{1-p_{c}}$$

where *P_o_* and *P_c_* are the observed agreement and the expected agreement, respectively.

The performance of classification based on the kappa coefficient can be classified as: poor agreement (<0), slight agreement (0–0.2), fair agreement (0.21–0.40), moderate agreement (0.41–0.60), good agreement (0.61–0.80), and very good agreement (0.81–1) [35]

## 3. Results

### 3.1. Pattern Classification Distribution

A total of 16,376 samples (from 11,373 patients) with images stored in the NOVA View instrument were categorized as positive IIF pattern(s) samples (Table 1). The samples of pattern classification distribution were as follows: 8663 (24.9%) homogenous, 395 (1.1%) nuclear dense fine speckled, 903 (2.6%) centromere, 14,992 (43.1%) nuclear speckled, 752 (2.2%) discrete nuclear dots, 1016 (2.9%) nuclear nucleolar, 126 (0.4%) cytoplasmic fibrillar, 2515 (7.2%) cytoplasmic speckled, 375 (1.1%) cytoplasmic AMA, 55 (0.2%) cytoplasmic Golgi, 54 (0.2%) cytoplasmic rods and rings. Moreover, we obtained an additional 121 cytoplasmic fibrillar, 74 cytoplasmic AMA, 406 cytoplasmic Golgi, and 624 cytoplasmic rods and rings images using the imbalanced data correction method described above.

### 3.2. Model Training and Comparison of Different Models on Testing Data 1

The average running time to analyze an ANA image is about 0.57 s on a GPU (NVIDIA TITAN V, 12GB RAM). The training time for our six transfer learning CNN models, including VGG19, ResNet50V2, DenseNet121, MobileNetV2, Xception, and InceptionResNetV2, takes about 85 h, 59 h, 39 h, 85 h, 46 h, and 72 h, respectively.

**Figure 4:**
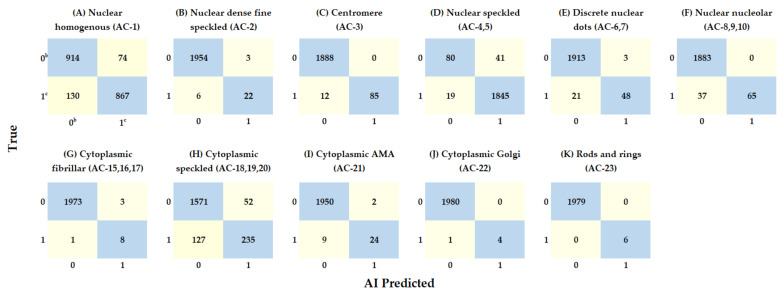
(**A**–**K**) Confusion matrices of the best performance proposed model (InceptionResNetV2) on sample prediction on Testing Data 1 ^a^. ^a^ Consists of 1985 samples with 6195 images ^b^ 0: negative, ^c^ 1: positive.

Table 2 shows the comparison among six state-of-the-art pre-trained models on Testing Data 1 which consists 1985 samples. The InceptionResNetV2 model achieved the highest F1 score (0.86) and the highest kappa (0.82). Therefore, we adopted the InceptionResNetV2 architecture via transfer learning technique as the proposed model in our study. Figure 3B shows the overall framework of ANA mixed patterns classification using the InceptionResNetV2 models. The details of the InceptionResNetV2 model performance for each classification on Testing Data 1 are presented by the confusion matrix in Figure 4.

### 3.3. Performance of the Best Performance Proposed Model on Testing Data 1 

Table 3 shows the performance obtained from the proposed model for the sample prediction on Testing Data 1, which consists of 1985 samples. The accuracy ranged from 0.91 to 1.00 (mean: 0.98). However, accuracy is potentially unhelpful for this asymmetric real-world clinical dataset in this work. We use F1 score to evaluate the models’ performance as a figure of merit on the testing data. This metric offers a more conservative view of model performance relative to accuracy when the class distribution is unequal. The precision, recall, and F1 score ranged from 0.73 to 1.00 (mean: 0.93), 0.64 to 1.00 (mean: 0.81), and 0.72 to 1.00 (mean: 0.86), respectively, varying across all of the competent-level pattern classifications. The top three patterns with highest F1 score were “cytoplasmic rods and rings” (1.00), nuclear speckled (0.99) and centromere (0.93). A comparison of consistency among experienced readers, the kappa value of each classification ranged from 0.67 to 1.00 (mean: 0.82), which means the proposed model achieved an almost perfect agreement with the experienced readers overall. The top three patterns with highest kappa values were “cytoplasmic rods and rings” (1.00), centromere (0.93), and cytoplasmic Golgi (0.89). It is worth mentioning that the F1 score improved from 0.29 to 1.0 after brightness data augmentation for the cytoplasmic rod and ring’s classification. However, the improvement may contribute to the correction of imbalanced data.

### 3.4. Inter-Observer Agreement (IOA) by Testing Data 2

Table 4 presents the results of IOA from five experienced readers (A1–A5), two new rheumatology fellows-in-training (F1, F2), and our proposed model (AI; InceptionResNetV2), which was evaluated by Testing Data 2 consists of 175 randomly selected samples with a total of 544 images. The average agreement among the five experienced readers for the eleven pattern classifications was 0.849 (κ), indicating almost perfect agreement. However, the average agreement between the five experienced readers and the two rheumatology fellows-in-training was 0.528 (κ), indicating moderate agreement. Overall, the average agreement between our proposed model (InceptionResNetV2) and five experienced readers reached 0.844 (κ), indicating almost perfect agreement. The results described above indicate that our proposed model outperformed beginners and might even be capable of replacing experienced experts.

## 4. Discussion

The results demonstrated that the consistency between five experienced readers could reach a Cohen’s kappa coefficient of 0.849 (Table 4). This means that by using the ICAP competent level classification standards, the consistency with human reading reached an almost perfect agreement. Furthermore, the performance of the proposed model (InceptionResNetV2) achieved almost perfect agreement (κ = 0.844) with experienced medical technologists. In contrast, the agreement between two new beginners and experienced medical examiners was only 0.528. The results shed light on a deep learning method could potentially save time training beginners in laboratories. 

Although there have been many related studies conducted in this field, including some that reached high accuracy [8,9,15,36,37], the classifications were not in accordance with well-established ICAP standards [1,2]. Another issue is that the question to be answered in previous studies was, “Which classification should this image belong to?” which was a “single choice question.” Most of the image datasets used were specifically prescreened well-defined IIF patterns with no mixed patterns. However, the ANA classification question in the real world is “what pattern(s) appear in this image?” which is a more complex “multiple choice question.” Every pattern must be identified, so that the follow-up test to suspected autoantibody or clinically relevant information will not be missed. However, it is worth noting there is no consensus on mixed patterns interpretation currently [2], particularly for weak cytoplasmic speckled staining images, which are very subjective when it comes to categorizing cytoplasmic staining as positive or negative. In these cases, there will be inconsistencies between readings performed by human experts. Thus, we used a consistency test instead of accuracy to evaluate the performance of machine learning models. Our results are not suitable for comparison with previous research because different questions and evaluation methods were used.

In previous studies, most methods performed better for pattern classification at specimen-level HEp-2 image than cell-level [12,38]. This is because a correct pattern of an image does not require a correct pattern of all cells present, but only a correct predominant cell. For the above reasons, we used the image as a whole rather than segmented cells for training our machine learning model. This also meets the interpretation rules of ICAP that state final patterns should be determined by integrating the features of interphase and mitotic metaphase cells.

**Figure 5:**
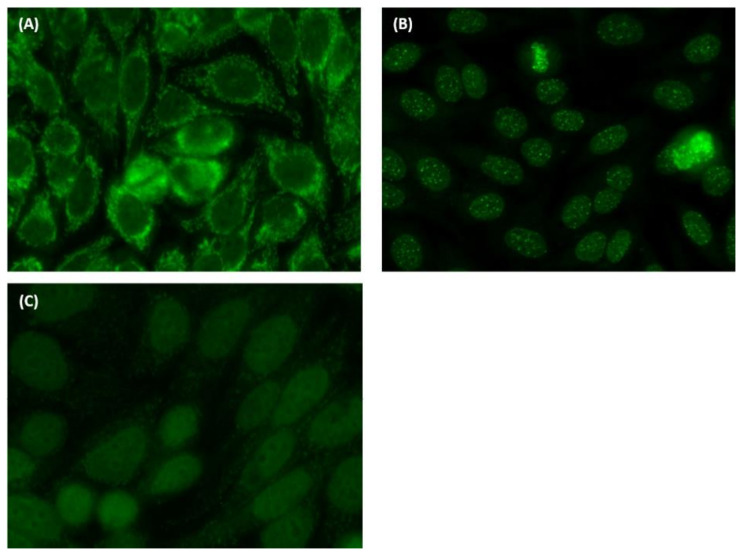
Examples of mixed pattern images that commonly leading to inconsistent reading. (**A**) High consistency of cytoplasmic staining pattern, but is inconsistent if there is a “nuclear fine speckled” pattern. (**B**) High consistency of nuclear centromere pattern, but is inconsistent if there is a “nuclear fine speckled” pattern. (**C**) High consistency of nuclear fine speckled pattern, but is inconsistent if there is a “cytoplasmic speckled” pattern, especially in very weak cytoplasmic staining images.

The two most frequent pattern classifications in this study were nuclear homogenous and nuclear speckled, which were compatible with the findings of Vermeersch et al. [39]. In our study, satisfactory agreements were found for the centromere, discrete nuclear dots, cytoplasmic fibrillar, cytoplasmic Golgi, cytoplasmic rods and rings, which consist of special and apparent features compared to other patterns. In contrast, we found lower agreements for nuclear speckled, cytoplasmic speckled, and cytoplasmic AMA patterns compared to other patterns. We propose several reasons to explain these findings, as follows. When reading mixed pattern images, the following situations are particularly subjectively interpreted and cause inter-observer variation. First, discriminate nuclear speckled positive/negative when strong cytoplasmic staining (Figure 5A) or centromere patterns are present (Figure 5B). Second, discriminate cytoplasmic patterns are positive/negative with weak cytoplasmic fluorescence intensity (Figure 5C). In fact, by using digital image reading, the brightness of the computer or smartphone screen may impact human interpretation. Therefore, more consensus is needed for the above situation.

Currently, at least seven commercial systems for the automated reading of ANA IIF are available (Table 5): Aklides, (Medipan, Dahlewitz, Germany), EUROPattern (Euroimmun AG, Luebeck, Germany), Helios (Aesku Diagnostics, Wendelsheim, Germany), Image Navigator (ImmunoConcepts, Sacramento, CA, USA), NOVA View (Inova Diagnostics, San Diego, CA, USA), Zenit G-Sight (A. Menarini Diagnostics, Florence, Italy), Cytospot (Autoimmun Diagnostika, Strassberg, Germany). All of these systems can classify samples as positive or negative. IIF pattern numbers identified by the currently available automated systems ranged from five to eight, which did not reach the standard of 11 classifications of ICA competent level report. Besides, as for our acknowledgment and available data, it seems like only the EUROPattern claims to identify mixed patterns.

There were some limitations in this study and the proposed model. First, no external validation was done. The model in this work was trained using images generated by the same instrument in a single hospital and not validated by images produced by instruments from the same manufacturer in other hospitals. Future initiatives should consider this issue by adopting large-scale datasets, including images obtained by acquisition devices, equipment, or instruments from different diagnostic centers. Second, for each sample, the restricted images captured by our instrument may not wholly represent what humans see by microscopic reading. If metaphase cells in the field of views captured by the instrument’s camera are atypical, such as mitosis in prophase, anaphase or telophase, the pattern cannot be correctly interpreted, particularly for the homogenous pattern, which needs typical metaphase cells for interpretation. Third, considering the workflow, we did not routinely perform an endpoint titer for every pattern in our laboratory and the proposed model could not provide titer information. However, ANA by IIF is not a quantitative assay [41], and the value of cytoplasmic titer for clinical application is still unknown which further research is needed.

In summary, our research showed that with sufficient image data labeled appropriately by experts, excellent consistency performance could be achieved by machine learning methods. In future research, there are several areas worth pursuing. First, harmonize the consistency of interpretation among different laboratories, and gather large and high-quality image datasets from multiple medical centers to establish a perfect machine learning model. Second, promote the consensus of mixed pattern interpretation, especially the judgment of positive and negative cytoplasmic patterns. Third, work on automated HEp-2 cell expert-level pattern recognition, which requires longer learning time and relies more heavily on experienced experts. A successful automatic interpretation system and a privacy-focused cloud platform might solve the problem of insufficient experts, and more importantly, it would shorten the time of manual interpretation, thereby improving the efficiency of laboratories.

## 5. Conclusions 

This study demonstrated that a publicly available pre-trained CNN model applying to a large clinically useful HEp-2 dataset with fine-tuning could achieve exceptionally high agreement of experienced human reading for ANA patterns, even without a novel image-processing technique used. In particular, it meets the standards of ICAP competent Level 11 classifications and mixed pattern recognition with high potential for immunologic laboratory automated diagnostic support.

## Figures and Tables

**Table 1:** ICAP classification distribution, image number for machine learning, analysis of relevant follow-up test in the study dataset.

ICAP Competent Level Classification	Patient Number *n* = 25,798 ^a^ (%)	Sample Number *n* = 34,756(%)	Image Number for Machine Learning ^b^			Relevant Follow-Up Test Available in Our Laboratory	Patients Receiving Follow-Up Test ^c^/Positive Number (%)
			Training	Validation	Testing ^d^		
Nuclear homogeneous AC-1	6293 (24.3)	8663 (24.9)	21,596	5398	3106	anti-DsDNA ^e^	2160/470 (21.8)
						anti-Histone ^e^	115/30 (26.1)
Nuclear dense fine speckled AC-2	247 (1.0)	395 (1.1)	981	244	95	anti-DFS ^f,g^	31/29 (93.5)
Centromere AC-3	545 (2.1)	903 (2.6)	2239	561	307	anti-CENP ^f^	302/291 (96.4)
Nuclear speckled AC-4, 5	10,632 (41.0)	14,992 (43.1)	37,577	9396	5813	AC4: anti-SSA/SSB ^f^	5379/1542 (28.7)
						AC4: anti-Mi2, TIF, NXP2, SAE1, Ku ^g^	1035/272 (26.3)
						AC5: anti-Sm/RNP ^f^	244/144 (59.0)
Discrete nuclear dots AC-6, 7	446 (1.7)	752 (2.2)	1882	470	213	anti-NXP2 ^g^	25/2 (8)
Nuclear nucleolar AC-8, 9, 10	782 (3.0)	1016 (2.9)	2524	630	326	anti-PM-Scl 75/100 ^g^ or Scl-70 ^f^	187/49 (26.2)
Cytoplasmic fibrillar AC-15, 16, 17	107 (0.4)	126 (0.4)	410	103	28	none	none
Cytoplasmic speckled AC-18, 19, 20	2005 (7.7)	2515 (7.2)	6380	1595	1122	anti-PL-7, PL-12, Jo-1, SRP, EJ, OJ, MDA5 ^g^	152/ 74 (48.7)
						anti-Jo-1 ^f^	90/13 (14.4)
						anti-Ribosomal P ^e^	26/10 (38.5)
Cytoplasmic AMA AC-21	264 (1.0)	375 (1.1)	980	245	105	anti-Mitochondria ^h^	88/52 (59.1)
						anti-Mitochondria-M2 ^e^	10/9 (90)
Cytoplasmic Golgi AC-22	37 (0.1)	55 (0.2)	463	116	15	none	none
Cytoplasmic rods and rings AC-23	44 (0.2)	54 (0.2)	635	159	18	none	none
Positive IIF pattern(s)	11,373 (44.1)	16,376 (47.1)					
AC-0	15,299 (59.3)	18,380 (52.9)					

ICAP, International Consensus on Antinuclear Antibody Patterns; IIF, indirect immunofluorescence. ^a^ A few patients have more than one antinuclear antibody (ANA) test at different timings with variable pattern reports. ^b^ Each sample generated 3–8 images by NOVA View instrument. ^c^ Determined by clinical decision. ^d^ From Testing Data 1. ^e^ Provided by enzyme-linked immunosorbent assay. ^f^ Provided by fluorescence enzyme immunoassay. ^g^ Provided by immunoblot. ^h^ Provided by IIF.

**Table 2:** Comparison of performance on sample prediction among six pre-trained models on Testing Data 1 ^a^.

Models	F1 Score	Kappa
InceptionResNetV2	0.86	0.82
MobileNetV2	0.81	0.76
Xception	0.78	0.74
VGG19	0.78	0.73
ResNet50V2	0.73	0.68
DenseNet121	0.68	0.63

^a^ Consists of 1985 samples.

**Table 3:** Sample prediction performance of the best performance proposed model (InceptionResNetV2) on Testing Data 1 ^a^.

	Accuracy	Precision	Recall	F1 Score	Kappa
Nuclear homogeneous (AC-1)	0.90	0.92	0.87	0.89	0.79
Nuclear dense fine speckled (AC-2)	1.00	0.88	0.79	0.83	0.83
Centromere (AC-3)	0.99	1.00	0.88	0.93	0.93
Nuclear speckled (AC-4, 5)	0.97	0.98	0.99	0.99	0.74
Discrete nuclear dots (AC-6, 7)	0.99	0.94	0.70	0.80	0.79
Nuclear nucleolar (AC-8, 9, 10)	0.98	1.00	0.64	0.78	0.77
Cytoplasmic fibrillar (AC-15, 16, 17)	1.00	0.73	0.89	0.80	0.80
Cytoplasmic speckled (AC-18, 19, 20)	0.91	0.82	0.65	0.72	0.67
Cytoplasmic AMA (AC-21)	0.99	0.92	0.73	0.81	0.81
Cytoplasmic Golgi (AC-22)	1.00	1.00	0.80	0.89	0.89
Cytoplasmic rods and rings (AC-23)	1.00	1.00	1.00	1.00	1.00
Mean	0.98	0.93	0.81	0.86	0.82

^a^ Consists of 1985 samples.

**Table 4:** Inter-observer agreement (Cohen’s kappa) on Testing Data 2 among five experienced medical technologists (A1–A5), two rheumatologic fellows-in-training (F1–F2), and the InceptionResNetV2 AI model (AI).

	Nuclear Homogeneous	Nuclear Dense Fine Speckled	Centromere	Nuclear Speckled	Discrete Nuclear Dots	Nuclear Nucleolar	Cytoplasmic Fibrillar	Cytoplasmic Speckled	Cytoplasmic Reticular/AMA	Polar/Golgi-like	Rods and Rings	Average
Pairwise comparison between five experienced medical technologists												
A1 vs A2	0.872	0.656	1.000	0.720	0.913	0.949	0.739	0.524	0.723	0.903	1.000	0.818
A1 vs A3	0.856	0.719	1.000	0.796	0.870	0.788	1.000	0.679	0.766	0.953	1.000	0.857
A1 vs A4	0.859	0.656	0.982	0.738	0.841	0.953	0.791	0.698	0.791	0.911	1.000	0.838
A1 vs A5	0.850	0.794	1.000	0.813	1.000	0.800	0.920	0.776	0.738	1.000	1.000	0.881
A2 vs A3	0.856	0.791	1.000	0.823	0.849	0.833	0.739	0.759	0.953	0.949	1.000	0.868
A2 vs A4	0.910	0.869	0.982	0.789	0.823	0.903	0.833	0.712	0.788	0.903	1.000	0.865
A2 vs A5	0.825	0.851	1.000	0.677	0.913	0.752	0.815	0.556	0.646	0.903	1.000	0.813
A3 vs A4	0.921	0.930	0.982	0.793	0.766	0.848	0.791	0.761	0.722	0.860	1.000	0.852
A3 vs A5	0.835	0.920	1.000	0.751	0.870	0.700	0.920	0.682	0.682	0.953	1.000	0.847
A4 vs A5	0.837	0.851	0.982	0.670	0.841	0.845	0.869	0.788	0.815	0.911	1.000	0.855
Average	0.862	0.804	0.993	0.757	0.869	0.837	0.842	0.694	0.762	0.925	1.000	0.849
Pairwise comparison between five experienced medical technologists and two rheumatologic fellows-in-training												
A1 vs F1	0.764	0.330	0.982	0.166	0.676	0.692	0.718	0.465	0.652	0.953	0.821	0.656
A1 vs F2	0.723	0.532	0.945	0.096	0.445	0.000	0.235	0.526	0.033	0.246	0.949	0.430
A2 vs F1	0.738	0.344	0.982	0.114	0.771	0.739	0.515	0.642	0.788	0.949	0.821	0.673
A2 vs F2	0.698	0.513	0.945	0.122	0.450	0.000	0.165	0.448	-0.008	0.289	0.949	0.416
A3 vs F1	0.771	0.473	0.982	0.139	0.719	0.791	0.718	0.592	0.722	0.903	0.821	0.694
A3 vs F2	0.731	0.405	0.945	0.132	0.478	0.000	0.235	0.537	0.000	0.266	0.949	0.425
A4 vs F1	0.750	0.445	0.964	0.114	0.697	0.651	0.555	0.673	0.738	0.953	0.821	0.669
A4 vs F2	0.685	0.373	0.927	0.076	0.408	0.000	0.180	0.524	0.024	0.246	0.949	0.399
A5 vs F1	0.695	0.393	0.982	0.111	0.676	0.522	0.655	0.564	0.567	0.953	0.821	0.631
A5 vs F2	0.680	0.441	0.945	0.053	0.445	0.000	0.215	0.590	0.101	0.246	0.949	0.424
F1 vs F2	0.640	0.156	0.925	0.472	0.454	0.000	0.258	0.453	-0.065	0.266	0.768	0.393
Average	0.716	0.400	0.957	0.145	0.565	0.309	0.404	0.547	0.323	0.570	0.874	0.528
Pairwise comparison between five experienced-certified medical technologists and InceptionResNetV2 AI model												
A1 vs AI	0.795	0.719	1.000	0.772	0.833	0.692	1.000	0.698	0.791	0.848	1.000	0.832
A2 vs AI	0.847	0.930	1.000	0.899	0.815	0.739	0.739	0.739	0.683	0.944	1.000	0.849
A3 vs AI	0.830	0.851	1.000	0.851	0.823	0.791	1.000	0.761	0.722	0.894	1.000	0.866
A4 vs AI	0.833	0.930	0.982	0.817	0.794	0.651	0.791	0.828	0.869	0.848	1.000	0.849
A5 vs AI	0.775	0.920	1.000	0.727	0.833	0.522	0.920	0.728	0.815	0.848	1.000	0.826
Average	0.816	0.870	0.996	0.813	0.820	0.679	0.890	0.751	0.776	0.876	1.000	0.844
Pairwise comparison between two rheumatologic fellows-in-training and InceptionResNetV2 AI model												
F1 vs AI	0.738	0.367	0.982	0.118	0.685	1.000	0.718	0.623	0.607	0.894	0.821	0.687
F2 vs AI	0.698	0.405	0.945	0.092	0.384	0.000	0.235	0.496	0.024	0.316	0.949	0.413
Average	0.718	0.386	0.964	0.105	0.535	0.500	0.477	0.560	0.316	0.605	0.885	0.550

**Table 5:** Characteristics of automated readers for Hep-2 image patterns identified by ANA IIF testing ^a^.

Automated System	No. of Patterns Recognized ^b^	Mixed Pattern Recognition
Aklides	6	only very few predefined
EUROpattern	8	yes
Helios	7	no
Image Navigator	positive/negative	no
NOVA View	5	no
Zenit G-sight	5	no
Cytospot	positive/negative	no
Our proposed model	11	yes

^a^ Adapted from [40], with modification. ^b^ Aklides: cytoplasmic, homogeneous, speckled, nucleolar, centromere, and multiple nuclear dots patterns; EUROPattern: homogeneous, dense fine speckled, speckled, nucleolar, centromere, nuclear dots, nuclear membrane, and cytoplasmic patterns; Helios: centromere, cytoplasmic, homogeneous, nuclear membrane, nuclear dots, nucleolar, and speckled (granular) patterns; NOVA View: homogeneous, speckled, centromere, nucleolar, and nuclear dot patterns; Zenit G-Sight: homogeneous, nucleolar, speckled, centromere, and mitochondrial patterns; our proposed model: homogeneous, nuclear dense fine speckled, centromere, nuclear speckled, discrete nuclear dots, nucleolar, cytoplasmic fibrillar, cytoplasmic speckled, cytoplasmic reticular/anti-mitochondrion (AMA), polar/Golgi-like, rods and rings patterns.

## Data Availability

Raw data are available from the corresponding author on reasonable request.
